# A prokinetic agent DA-9701 formulated with *Corydalis tuber* and *Pharbitidis semen* improves nausea in migraine: A prospective observational study

**DOI:** 10.1097/MD.0000000000047623

**Published:** 2026-02-13

**Authors:** Hyoshin Son, Seunghyun Lee, Mi Ji Lee, Manho Kim

**Affiliations:** a Department of Neurology, Eunpyeong St. Mary’s Hospital, The Catholic University of Korea, Seoul, South Korea; b Medical School, Jeonbuk National University, Jeonju, South Korea; c Department of Neurology, Seoul National University Hospital, Seoul, Korea; d Department of Neurology, Seoul National University College of Medicine, Seoul, Korea.

**Keywords:** frequency, headache, migraine, nausea, prokinetic, side effects

## Abstract

Nausea is common in migraines and is one of the most prevalent neurological disorders. Although migraine prophylaxis is known to reduce nausea, the impact of nausea treatment on migraine headache frequency remains unclear. DA-9701, a prokinetic derived from *Corydalis tuber* and *Pharbitidis semen*, offers serotonin 5-HT1A/5-HT4 agonism and D2 antagonism, with a reduced risk of adverse effects associated with traditional D2 antagonists. This study assessed the efficacy and short-term tolerability of DA-9701 in treating nausea and migraine and evaluated its secondary effect on reducing the frequency of migraine headaches. A cohort of 110 migraineurs with nausea received DA-9701 for 1 month. Using a headache diary, the monthly frequency of headache and nausea was recorded during a 1-month baseline and a 1-month treatment period. Changes in the number of nausea days per month were measured as the primary outcome. Changes in headache days and days requiring acute rescue medication were assessed as secondary outcomes using the DIEPSS. Further analyses were performed according to the migraine subtypes EM and CM. Nausea days decreased by 53.7% (−4.65 days; 95% confidence interval (CI) −10.66 to −4.66 days; *P* <.001). Headache days were reduced by 52.2% (−8.18 days; 95% CI −15.35 to −11.19 days;*P* <.001), and rescue medication days by 44.8% (−3.46 days; 95% CI −6.75 to −1.00 days; *P* <.001). In the CM group, changes in headache days were more prominent than those in nausea days (−13.47 vs −7.12 days; *P* <.001). No EPS or other clinically relevant adverse events were observed. DA-9701 was well tolerated and reduced both nausea and headache in migraineurs. The CM data suggest a potential independent prophylactic effect of DA-9701 on migraine headache. Larger randomized controlled trials are warranted.

## 1. Introduction

Migraine, the most prevalent neurological disorder, affects 10% to 15% of the global population.^[1]^ In South Korea alone, approximately ten million individuals are afflicted with this condition In South Korea alone.^[2]^ In the United States, government health surveillance indicates that migraines significantly hamper daily life, representing a substantial health burden.^[3]^ Patients frequently experience not only debilitating headaches but also accompanying symptoms, such as nausea and vomiting, often leading to emergency room visits.^[3,4]^

A notable interconnection exists between migraines and digestive disorders. More than 60% of individuals with functional dyspepsia are also affected by migraine.^[5]^ Furthermore, the odds ratio of experiencing migraine is 1.6 for those suffering from irritable bowel syndrome (IBS).^[6]^ Nausea, the most frequently associated symptom, is included in the diagnostic criteria for migraine.^[7]^

Interestingly, episodes of vomiting have been observed to alleviate acute migraine attacks, suggesting a gastrointestinal (GI) contribution to migraine pathophysiology.^[8]^ However, the effectiveness of migraine prophylaxis in mitigating nausea also suggests a possible link with central nervous system hypersensitivity.

Management of nausea often involves the use of prokinetics or anti^[9,10]^ Medications that inhibit dopaminergic D2 receptors such as metoclopramide are commonly prescribed. Nevertheless, these treatments can lead to undesirable side effects, including extrapyramidal symptoms (EPS) such as acute dystonia, akathisia, or parkinsonism, and hyperprolactinemia-related adverse effects, including galactorrhea and menstrual irregularities, which may limit their continued use.^[11]^

*Corydalis tuber* (the dried tuber of *Corydalis yanhusuo*) has traditionally been used in East Asian medicine for gastrointestinal discomfort and analgesia, while *Pharbitidis semen* (the seeds of *Pharbitis nil*) has been utilized as a purgative and for digestive symptoms. DA-9701 is a standardized botanical extract formulated with *Corydalis tuber* and *Pharbitidis semen* and developed as a prokinetic agent for functional gastrointestinal disorders.^[12,13]^ DA-9701 exhibits agonistic effects on serotonin 5-HT1A and 5-HT4 receptors while antagonizing D2 receptors.^[13]^ A 2-week observational study involving 100 subjects reported a low incidence of acute EPS and stable prolactin levels, suggesting a favorable safety profile. The limited permeability of DA-9701 through the blood–brain barrier is thought to contribute to these outcomes.^[14,15]^ Moreover, a placebo-controlled 4-week trial in patients with IBS and functional dyspepsia overlap did not report significant adverse events.^[16]^

Despite the acknowledged safety profile in GI disorders, its efficacy and safety of DA-9701 in managing nausea associated with migraine remain unclear. Moreover, it is yet to be determined whether alleviating nausea can subsequently reduce the frequency or intensity of migraine headache.

**Figure 1.**
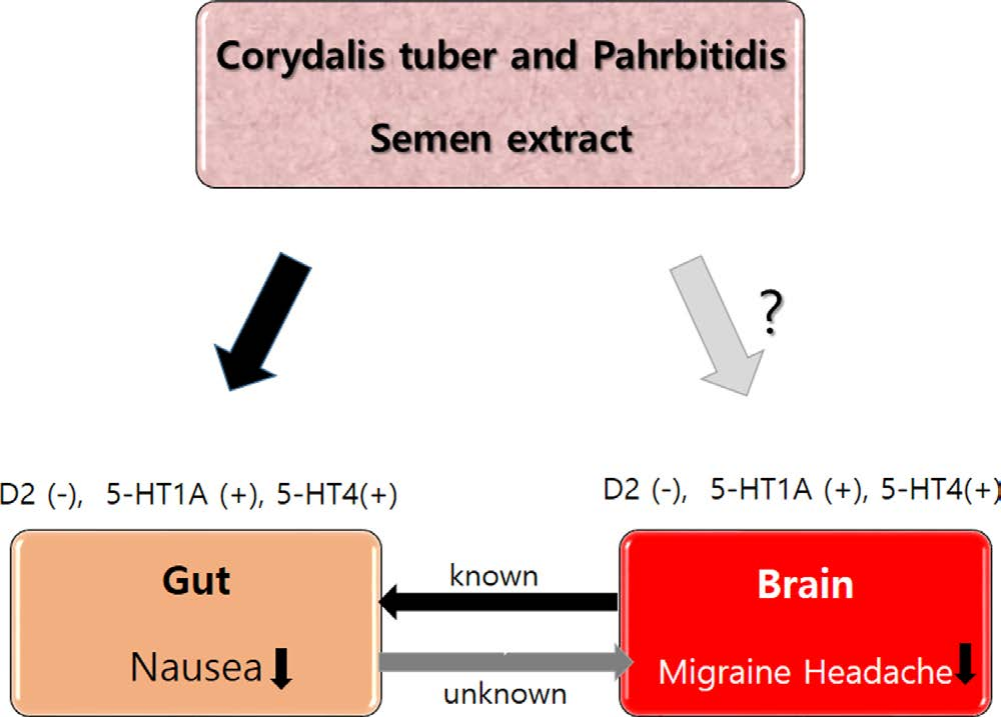
Effect DA-9701 on the gastrointestinal system and a proposed association in the nervous system. Formulated Corydalis tuber and Pahrbitidis semen (DA-9701) reduce nausea through a prokinetic effect mediated by the inhibition of D2 and agonism of 5-HT1A and 5-HT4 receptors. Migraine is frequently associated with nausea, and reducing headaches is known to decrease nausea. However, it is unknown (gray arrow: unknown) whether reducing nausea can decrease migraine headaches. DA-9701 may have a direct or indirect effect on migraine headaches (gray arrow). 5-HT1A = 5-hyroxytryptamine type 1A, 5-HT4 = 5-hyroxytryptamine type 4, D2 = dopamine subtype 2, DA-9701 = a standardized botanical prokinetic extract derived from *Corydalis tuber* and *Pharbitidis semen*.

This study aimed to evaluate the efficacy of DA-9701 in treating nausea in patients with migraine. Furthermore, we explored whether the reduction in nausea was accompanied by a decrease in migraine headache frequency (Fig. 1).

## 2. Methods

### 2.1. Subjects

A total of 110 migraine patients experiencing nausea were consecutively recruited from the outpatient neurology clinic between March 2021 and February 2022. Migraine was diagnosed according to the International Classification of Headache Disorder the 3^rd^ edition beta (ICHD-IIIβ) criteria. The cohort comprised 8 men and 102 women, with an average age of 50.6 ± 15.7 years (mean ± standard deviation; 44.8 ± 17.8 years for men and 51.0 ± 15.5 years for women, *P* = .34). Demographic data collected included age, sex, body mass index (BMI), migraine subtype, episodic migraine versus chronic migraine (EM versus CM), and duration of migraine history. The inclusion and exclusion criteria are presented in Table 1. There was no drop-out.

**Table 1:** The inclusion and exclusion criteria.

Inclusion criteria	Exclusion criteria
① Migraineurs associated with nausea or vomiting ② Those who agreed to participate in this study ③ Adults aged 19 to 85 yr ④ Subjects who meet the diagnostic criteria of migraine according to the ICHD-IIIβ ⑤ Subjects with migraine onset at least 3 mo and with moderate intensity (NRS 4–6) of 4 d or more on average in the last 3 mo ⑥ Subjects who had nausea or vomiting at least 3 mo ⑦ Who can read and understand the migraine diagnostic questionnaire ⑧ Subjects who agreed to take DA-9701 with written consent form	① Subjects whose headache are believed to be caused by secondary causes ② The headache developed at the age of 50 or older ③ Subjects with a history of renal or hepatic failure ④ Pregnant or women with lactation ⑤ Subjects currently participating in other clinical trials under R&D in addition to this study ⑥ When the researcher judges that participation in this study threatens the well-being of the subject or results in an unbelievable evaluation at the end of the study ⑦ Subjects who have started migraine prevention treatment within the last 4 weeks (including propranolol, tricyclic antidepressant, valproic acid, topiramate, BOTOX, or other CGRP antagonists) ⑧ Subjects with a history of alcohol and drug abuse. ⑨ Subjects taking anticholine and papa milk-containing drugs ⑩ Subject with Parkinson disease, other extrapyramidal disorders, or those taking drugs that can cause extrapyramidal symptoms. ⑪ Subject receiving medicine at the gastroenterology department or those who had a history of surgery. ⑫ Subjects with history of allergic reaction to DA-9701 ⑬ Subjects with arrhythmia or long QT syndrome

For recruitment of migraineurs with nausea, inclusion of migraine was met the international guideline. Legal definition of adult is from 19 years in South Korea. Exclusion criteria contained conditions that interfere the interpretation of data and those who are not likely diagnosed as migraine. DA-9701 is commercially available by prescription in South Korea since 2011, thus, subjects could have past medical histories of taking DA-9701, and were able to know whether they are allergic or not.

BOTOX = botulinum toxin type A, CGRP = calcitonin gene related peptide, DA-9701 = a standardized botanical prokinetic extract derived from *Corydalis tuber* and *Pharbitidis semen*, ICHD IIIβ = International Classification of Headache Disorder version III beta, NRS = numeric rating scale, R&D = research and development.

The exclusion criterion previously described as “when the researcher judges that participation in this study threatens the well-being of the subject or results in an unbelievable evaluation at the end of the study” was operationalized as follows: participation was excluded when the investigator judged that it could threaten the well-being of the subject (e.g., severe uncontrolled psychiatric illness, active suicidal ideation, or unstable medical conditions such as decompensated cardiac or hepatic disease), or when reliable diary recording was deemed unlikely despite instruction (e.g., cognitive impairment, inability to understand the headache diary, or repeated noncompliance during a run-in period).

### 2.2. DA-9701, study design and protocol

DA-9701 (Motilitone; Dong-A ST Co., Ltd., Seoul, South Korea), a prokinetic agent approved by the Ministry of Food and Drug Safety and prescribed since 2011, was the focus of this study. Patients who agreed to start this medication and participated in the study were included. DA-9701 was prescribed at 90 mg/day, divided into 3 doses, over 1 month following the Ministry of Food and Drug Safety guidelines.

During the baseline period (one month prior to starting DA-9701), participants recorded the frequency of nausea/vomiting and headache days in a daily diary. Days requiring acute rescue medication were also noted. The same data were collected over 1 month during DA-9701 administration. Safety profiles were assessed via telephone interviews at the 2-week mark and in-person interviews at the clinic at the end of the month. For participants unable to visit due to reasons such as Coronavirus disease 2019 (COVID-19) quarantine, diary data were reviewed, and missing or unclear entries were clarified via phone. The use of acute rescue medications to manage migraine attacks was permitted during the study period.

Participants were instructed to complete the diary daily. The diary entries were reviewed at each clinic visit and during scheduled telephone interviews. Any missing or unclear entries were clarified retrospectively. If a day could not be reliably reconstructed, it was marked as missing and excluded from frequency calculations. In this cohort, all 110 enrolled participants provided usable diary data over both the baseline and DA-9701 periods, and no subject was excluded because of excessive missing entries.

The study protocol was approved by the Institutional Review Board of Seoul National University Hospital (IRB No. 2011-145-1175) and all participants provided written informed consent in accordance with the Declaration of Helsinki.

### 2.3. Endpoints and assessment

A diary was used to track the frequency of the episodes of nausea and vomiting. Vomiting was categorized under “nausea” if it was preceded by nausea. Instances of nausea, headache, or acute treatment occurring more than twice in a day were recorded as “one day.” Frequencies were calculated as “days per month” in accordance with the guidelines of the International Headache Society.

Frequency data from diaries were validated during clinic visits and phone interviews. Adverse events, including EPS, were assessed using the Drug-Induced Extrapyramidal Symptoms Scale (DIEPSS). The primary endpoint was the change in the frequency of nausea between baseline and DA-9701 periods. The secondary endpoints included changes in headache days and the use of acute rescue medications.

### 2.4. Subgroup classification

Participants were further analyzed based on demographic differences and migraine subtypes as per ICHD-IIIβ: EM, characterized by fewer than 15 headache days per month, and CM, defined as 15 or more headache days per month, with at least 8 of those days exhibiting migraine symptoms for a duration of 3 months or more.

### 2.5. Statistical analysis

The normality of continuous variables was assessed using the Shapiro–Wilk test. When the normality assumption was violated, we additionally confirmed the robustness by applying the Wilcoxon signed-rank test as a nonparametric alternative to the paired t-test. As the results of the parametric and nonparametric tests were concordant, we reported the *P*-values from the paired *t*-tests for simplicity. Paired t-tests were conducted for the primary and secondary endpoints. McNemar test was applied to nominal variables.

For ratio comparisons, the formula “(difference in days between baseline and DA-9701 period)/(days during baseline period)” was used. Pearson correlation analysis was performed to explore the relationships among variables, such as age and BMI. Among the collected demographic variables, age and BMI were selected a priori for correlation analyses because they have been implicated as potential risk factors for migraine chronification and disease burden.^[17]^ Statistical significance was indicated by * for *P*-values <.05 and ** for *P*-values <.001.

The primary endpoint was prespecified as the change in the number of nausea days per month. The secondary endpoints included changes in headache duration and days requiring acute rescue medication. Given the limited number of prespecified endpoints and the exploratory nature of subgroup analyses, no formal correction for multiple comparisons was applied; this is acknowledged as a limitation.

## 3. Results

### 3.1. Primary and secondary endpoints

**Figure 2.**
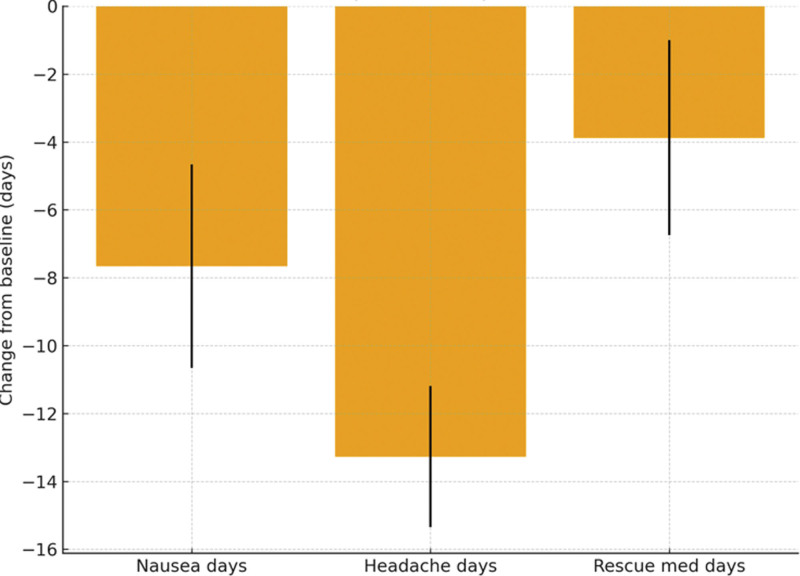
Changes in monthly days with nausea, headache, and acute rescue medication use after DA-9701 treatment. Mean absolute change in days per month for nausea, headache, and acute rescue medication use from the 1-month baseline period to the 1-mo DA-9701 treatment period in 110 migraine patients. Bars represent mean change (days/month), and error bars indicate 95% confidence intervals. Negative values reflect a reduction compared with baseline. DA-9701 = a standardized botanical prokinetic extract derived from *Corydalis tuber* and *Pharbitidis semen*.

The average monthly frequency of nausea during the baseline period was 8.64 ± 8.21 (mean ± SD) days, which reduced to 3.99 ± 5.85 days during the DA-9701 administration period, reflecting a 53.7% decrease (−4.65 days; 95% Confidence Interval (CI) −10.66 to −4.66 days; *P* <.001). Similarly, the frequency of headache episodes decreased by 52.2% (−8.18 days; 95% CI −15.35 to −11.19 days; *P* <.001), from 15.65 ± 9.94 7.47 ± 6.21 days/month (*P* <.001). The use of acute rescue medications also showed a substantial reduction by 44.8% (−3.46 days; 95% CI −6.75 to −1.00 days; *P* <.001), decreasing from 7.72 ± 9.23 to 4.26 ± 5.24 days/month (*P* <.001). These changes are illustrated in Figure 2.

### 3.2. Gender differences

A notable sex ratio difference was observed in this study (8 men vs 102 women). Although no statistically significant differences were detected between men and women in exploratory comparisons of nausea days, headache days, and acute rescue medication usage (Table 2), the small number of male participants (n = 8) precluded any reliable conclusions regarding sex-specific effects. Therefore, further analyses were conducted by pooling subjects together.

**Table 2:** Gender difference profiles for endpoints parameters.

	Men	Women	*P*-value
Nausea frequency at baseline	5.28	8.73	.79
Nausea frequency during DA-9701	4.38	4.04	.68
Headache frequency at baseline	16.6	15.6	.56
Headache frequency during DA-9701	6.62	7.54	.72
Acute rescue frequency at baseline	7.75	7.71	.99
Acute rescue frequency during DA-9701	3.64	4.31	.73

“Frequency” is the number of days per month. There were no significant differences observed.

DA-9701 = a standardized botanical prokinetic extract derived from *Corydalis tuber* and *Pharbitidis semen*.

### 3.3. Correlation analysis between parameters

Correlation analyses were conducted among endpoint parameters and factors, such as age and BMI. A significant association was found between the number of days of nausea and headache during the baseline period (*P* <.00001), which persisted in the DA-9701 period (*P* <.00001). The degree of change in these parameters was significantly correlated (*P* <.00001). However, no associations were observed between age or BMI and the primary or secondary endpoint parameters (Table 3).

**Table 3:** The correlations between parameters.

Parameters	Correlation coefficient	*P*-value
Baseline headache days ∝ baseline nausea days	0.49**	*P* <.00001
DA-9701 administration: headache days and ∝ nausea days	0.52**	*P* <.00001
Changes of headache days ∝ changes in nausea days	0.59**	*P* <.00001
Age ∝ change of headache days	0.08	*P* = .39
Age ∝ baseline headache days	0.09	*P* = .33
Age ∝ headache days with DA-9701	0.04	*P* = .65
BMI ∝ changes of headache days	0.03	*P* = .76

BMI ∝ body mass index, DA-9701 = a standardized botanical prokinetic extract derived from *Corydalis tuber* and *Pharbitidis semen*.

Age or BMI, one of factors that are related to migraine chronification, were not related to end point parameters in this study. The migraine headache and nausea are consistently correlated before and following the DA-9701 along with the degree of changes. Statistical significance was determined by **P <.001.

### 3.4. Subgroup analyses: episodic versus chronic migraine (EM vs CM)

#### 3.4.1. Demographic profiles

The study population comprised a higher proportion of women than of men. In the EM subgroup, there were 3 men and 55 women, whereas in the CM subgroup, there were 5 men and 47 women. The mean age for EM was 48.11 ± 15.85 years and for CM was 52.95 ± 15.23 years, indicating a trend toward older age in the CM group by an average of 4.84 years (*P* = .11). No significant difference in sex ratio was noted between the EM and CM groups.

#### 3.4.2. Endpoint parameters

##### 3.4.2.1. Baseline and DA-9701 periods

During the baseline period, the number of days of nausea, headache, and acute rescue medication use was higher in the CM group than in the EM group. This pattern persisted throughout the DA-9701 treatment period (Table 4).

**Table 4:** Subgroup analysis of endpoints parameters in EM and CM.

Migraine subtype	Baseline period		
	Frequency of nausea (days per month)	Frequency of headache (days per month)	Acute rescue medication (days per month)
EM	4.67	**6.91**	**5.09**
CM	12.65	24.58	10.21
*P*-value for CM > EM	6.22 E-07**	1.84 E-29**	0.0031*
	DA-9701 period		
EM	2.46	4.02	2.59
CM	5.53	11.11	6.08
*P*-value for CM >EM	0.0059*	1.40 E-09**	0.00086**

Bold values indicate *P* values for CM > EM.

E-07 = 10^−7^; E-29 = 10^−29^; E-9 = 10^−9^.

CM = chronic migraine, DA-9701 = a standardized botanical prokinetic extract derived from *Corydalis tuber* and *Pharbitidis semen*, EM = episodic migraine.

Endpoint parameters both in the baseline and following DA-9701 administration showed higher frequencies in CM than that of EM, suggesting reducing trend in frequency were in parallel both in EM and CM.

* *P* <.05.

** *P* <.001.

##### 3.4.2.2. Changes from baseline to the DA-9701 period

The reduction in acute rescue medication days was −2.50 days in the EM and −4.13 days in CM, which was not statistically significant between the subgroups (*P* = .41). However, the decrease in nausea days was significant, with −2.21 days in EM and −7.12 days in CM, resulting in a difference of 4.91 days (*P* = .001). The difference in headache days was also significant, with a 10.58-day greater reduction in CM than EM (−13.47 in CM vs −2.89 in EM; *P* = 9.55 × 10^−14^).

##### 3.4.2.3. Correlations among parameters

In the EM subgroup, these parameters were correlated in both the baseline and DA-9701 periods. In contrast, in the CM subgroup, no significant association was found between headache and nausea during the baseline period (*P* = .18). However, following DA-9701 administration, this subgroup exhibited a significant correlation between headaches and nausea (Table 5). Notably, the CM subgroup demonstrated a higher mean number of headache days than nausea days by 11.93 days, indicating a higher prevalence of headache days without accompanying nausea (Table 4).

**Table 5:** Correlation between parameters in EM and CM.

Nausea days ∝ headache days	Pearson correlation coefficients	
	EM	CM
Baseline period	0.49** (*P* = .000218)	0.18 (NS) (*P* = .176538)
DA-9701 period	0.70** (*P* = .00001)	0.43** **(*P* = .000877**)
Changing difference	0.49** (*P* = .00021)	0.41* (*P* = .00179)

Bold values indicate *P* values for CM > EM.

CM = chronic migraine, DA-9701 = a standardized botanical prokinetic extract derived from *Corydalis tuber* and *Pharbitidis semen*, EM = episodic migraine, NS = no significance.

CM did not show correlation at baseline period, however, by DA-9701 administration, correlation between nausea and headache. CM becomes significant, alike that of EM. (NS: no significant).

* *P* <.05.

** *P* <.001.

### 3.5. Safety profiles

Safety evaluations using the DIEPSS questionnaire, conducted approximately 2 and 4 weeks after the initiation of DA-9701, did not reveal any extrapyramidal symptoms. In addition, patients were systematically asked about galactorrhea, menstrual changes, gastrointestinal side effects (e.g., diarrhea, abdominal cramps, and altered bowel movements), and other new or worsening symptoms. No adverse events were reported during the 1-month treatment period.

## 4. Discussion

In this 1-month open-label study of 110 migraine patients with prominent nausea, DA-9701 was associated with a 53.7% reduction in nausea days, a 52.2% reduction in headache days, and a 44.8% reduction in rescue medication days. These changes were observed in both episodic and chronic migraines, with greater reductions observed in the CM subgroup. No extrapyramidal symptoms or other clinically relevant adverse events were observed during the study period.

Nausea and vomiting are frequently reported symptoms of gastrointestinal disorders and are notably associated with migraine, a widespread neurological condition.^[1–8]^ Although prokinetic agents and antiemetics have traditionally been employed to alleviate these distressing symptoms, their usage has been curtailed due to concerns over EPS and other adverse events associated with dopaminergic antagonism.^[9,11]^ These safety concerns have restricted their broader application in long-term or prophylactic treatment regimens.

Metoclopramide is a well-established antiemetic and prokinetic agent that is widely used in emergency departments for the acute treatment of migraine, particularly in patients who do not respond to conventional therapy.^[9–11]^ However, concerns over extrapyramidal symptoms and hyperprolactinemia limit its chronic or prophylactic use. DA-9701, which combines prokinetic and antiemetic properties with a low incidence of EPS and stable prolactin levels in prior gastrointestinal studies,^[12–16]^ may therefore represent a complementary option for migraine patients with prominent nausea, particularly in nonemergency outpatient settings.

The exact biological mechanism through which DA-9701 may contribute to the reduction in headache frequency remains unclear. While we observed decreases in both nausea and headache episodes, this did not necessarily establish a direct causal relationship between nausea management and migraine control. It is possible that DA-9701 modulates gut–brain interactions and autonomic function through its serotonergic and dopaminergic actions, with relatively limited penetration through the blood–brain barrier.^[13–15]^ An alternative, more speculative hypothesis is that peripheral desensitization – reducing nausea as a peripheral manifestation of migraine – could indirectly dampen central sensitization, a fundamental pathological process in migraine development.^[5,8]^ This concept is inspired by broader models of viscera–somatic convergence and gut–brain interaction in migraine and functional gastrointestinal disorders but has not been directly tested for DA-9701.

A demographic analysis of the study participants revealed a significantly high female representation, amounting to 92.7%, a finding that resonates with the broader understanding of a higher migraine prevalence in females, typically 3 times that in males.^[1,2,7]^ The exceptionally high female proportion in this study may partly reflect the clinical population and focus on migraine with prominent nausea. Although exploratory analyses did not show statistically significant gender-based differences in outcomes, the small number of male participants (n = 8) precluded any firm conclusions regarding sex-specific effects.

This study confirmed the previously reported peak age prevalence in migraine, with the average age in the CM group being approximately 4.84 years higher than that in the EM group.^[1,2]^ Contrarily, the anticipated trend of a transition from EM to CM was more prevalent among individuals with obesity, as no significant correlation with BMI was established.^[17]^ This deviation might stem from the characteristics of the study cohort, predominantly consisting of migraineurs with nausea, a condition that may lead to decreased appetite and, subsequently, a lower likelihood of weight gain.

Correlation analysis confirmed a close association between headache and nausea during both the baseline and DA-9701 periods, corroborating the diagnostic criteria for migraine without aura defined by the International Headache Society.^[7]^ However, the results indicated that CM patients experienced a relatively higher frequency of headache days without accompanying nausea than those with EM. This observation was substantiated by data showing that baseline headache days outnumbered nausea days in CM and that the co-occurrence of nausea and headache was not consistent in this group (Table 4). Subgroup analysis further revealed that DA-9701 significantly reduced headache days in CM, more so than nausea days, effectively aligning the CM phenotype more closely with that of EM, which was characterized by a pronounced correlation between nausea and headache. These findings suggest a potential independent prophylactic effect of DA-9701 on migraine headaches, particularly in the CM subgroup.

### 4.1. Limitations

This study had several limitations. First, its open-label, single-arm design without a control or placebo group precludes a definitive causal inference. Second, the findings rely on self-reported diaries and the retrospective clarification of some entries, which are subject to recall and reporting bias. Third, the observation period was relatively short (one month). Fourth, the marked sex imbalance (92.7% women) and single-center setting may limit generalizability. Fifth, although the mean reductions in nausea, headache, and rescue medication days were statistically significant, the standard deviations were large and, in some cases, approached the mean values, indicating substantial inter-individual variability. This suggests that, while many patients experienced robust improvement, others showed only modest or minimal responses. High variability reduces the precision of the effect size estimates and limits the generalizability of our findings. Finally, no formal correction for multiple comparisons was applied, which further emphasizes that the present results should be interpreted as exploratory and hypothesis generating.

## 5. Conclusions

DA-9701 can be a useful adjunctive treatment option for mitigating nausea and headache in individuals afflicted with migraine. In this cohort, DA-9701 was well tolerated and associated with substantial short-term reductions in both nausea and headache frequency, with particularly pronounced effects on chronic migraine. These preliminary findings highlight the need for further comprehensive randomized controlled studies and validation in larger cohorts to fully elucidate the therapeutic potential and mechanism of action of DA-9701 in migraine management.

## Author contributions

**Conceptualization:** Hyoshin Son, Manho Kim.

**Data curation:** Hyoshin Son.

**Formal analysis:** Hyoshin Son, Seunghyun Lee, Mi Ji Lee, Manho Kim.

**Funding acquisition:** Manho Kim.

**Supervision:** Manho Kim.

**Validation:** Mi Ji Lee.

**Writing – original draft:** Seunghyun Lee.

**Writing – review & editing:** Seunghyun Lee.
